# Concussion Subtype Identification With the Rivermead Post-concussion Symptoms Questionnaire

**DOI:** 10.3389/fneur.2018.01034

**Published:** 2018-12-03

**Authors:** Jun Maruta, Angela Lumba-Brown, Jamshid Ghajar

**Affiliations:** ^1^Brain Trauma Foundation, New York, NY, United States; ^2^Department of Neurosurgery, Stanford University School of Medicine, Stanford, CA, United States; ^3^Department of Rehabilitation and Human Performance, New York, NY, United States; ^4^Department of Emergency Medicine, Stanford University School of Medicine, Stanford, CA, United States

**Keywords:** mild traumatic brain injury (mTBI), epidemiology, cognitive, fatigue, vestibular, migraine, sleep

## Abstract

Classifying concussion in key subtypes according to presenting symptomatology at an early post-injury stage is an emerging approach that may allow prediction of clinical trajectories and delivery of targeted treatments. The Rivermead Post-concussion Symptoms Questionnaire (RPQ) is a simple, freely available, and widely used tool for assessment of the presence and severity of various post-concussion symptoms. We aimed to probe the prevalence among athletes of symptom classes associated with identified concussion phenotypes using the RPQ at baseline and acutely after a concussion. Participants of organized sports aged 12–30 years were baseline-assessed with the expectation that some would experience a concussion during the study period. Concussed athletes were re-assessed within 2 weeks of their injuries. The RPQ was supplemented with three specific questions and reworded for baseline assessment. A binomial test was used to contrast the prevalence of an attribute in the concussed cohort against the probability established by the baseline observation. Three thousand and eighty-eight athletes were baseline-assessed and eighty-nine were re-assessed post-concussion. All concussed athletes endorsed having some elevated symptoms in the RPQ, and such endorsements were more prevalent than those among normal athletes. Moderate-to-severe post-concussion symptoms of specific classes tended to be endorsed with few additional symptoms of other classes of similar intensities. Elevated symptoms detected with the RPQ within as short as 2 weeks after a concussion may help delineate patients' clinical subtypes and guide their treatment. Further refinement of symptom questionnaires and use of objective measures will be needed to properly populate the concussion subtype classification.

## Introduction

Concussions are heterogeneous—there is a broad consensus among experts that a one-size-fits-all approach to post-concussion management is ineffective (1–4). Subtype classification is an emerging effort within the concussion-related field. Based on clinical and anecdotal evidence, it has been suggested that the six clinical phenotypes of concussion described below may be profiled according to symptoms observed within about 1 week post-concussion (5, 6). Although this particular approach does not currently directly address the root-cause pathophysiology of symptoms as attempted in other approaches (7–9), such early-stage clinical profiling of concussion permits a targeted treatment by prioritizing management of most problematic issues while maximizing the initial impact on the patient's symptoms and impairment (6).

The six suggested profiles with partially overlapping symptoms are described as: (1) *cognitive-fatigue*, with symptoms of fatigue, decreased energy, non-specific headache, sleep disruption, or difficulty concentrating; (2) *vestibular*, with symptoms of dizziness, fogginess, nausea, feeling of being detached, or overstimulation in complex environments; (3) *oculomotor*, with symptoms of fatigue, distractibility, difficulties with visually based classes, pressure behind the eyes, or blurred or double vision; (4) *anxiety/mood*, with symptoms of anxiety, hypervigilance, feeling of being overwhelmed, sadness, or hopelessness; (5) *post-traumatic migraine*, with symptoms of headache with a pulsating quality associated with nausea, photosensitivity, or phonosensitivity; and (6) *cervical*, with symptoms of headache, neck pain, or numbness/tingling of the extremities.

We note that, among the six profiles, cervical attributes refer to extracranial origins and could be better conceptualized as an associated condition that affects recovery rather than a subtype of concussion. We also recognize that sleep disturbance is a condition that has emerged in the literature as affecting recovery from concussion (4, 10–12) and that it is considered to be a modifier in the recent update to this clinical profiling approach (6). We will thus distinguish sleep disturbance as a symptom class associated with concussion although not as characterizing a subtype of concussion.

The Rivermead Post-concussion Symptoms Questionnaire (RPQ) is a simple, freely available, and widely used tool for severity assessment of various post-concussion symptoms (13). The questionnaire was first of its kind when published in 1995, and has since been cited in hundreds of academic publications with an increasing trend. In its original form, the RPQ is based on a subjective five-scale rating of 0–4, relative to the premorbid levels, of 16 most commonly reported problems in the literature, with 0 indicating not experienced, and 4 severe. Respondents are also asked, if other difficulties exist, to rate on these additional symptoms. Here, we utilized slightly modified versions of the RPQ in a sports concussion study, in which more than 3,000 athletes were baseline-assessed and, in case of a subsequent concussion, re-assessed within 2 weeks post-injury.

Symptoms associated with concussion are not unique to concussion and can independently appear in normal settings (14, 15). The goal of this report is to probe the prevalence of symptom classes matching the current concussion profiling approach in a naturalistic athletic setting and during the acute post-concussion period using the RPQ. In such exploration, we also evaluate the ability of the RPQ to distinguish concussion clinical profiles.

## Methods

Data were collected as part of a larger research project on concussion with a particular focus on a normative characterization of eye movement performance in a variety of military and civilian groups. Collected data included demographic information, eye movements during visual tracking tasks, neurocognitive parameters, symptoms, and a history of previous head injuries. Some of the results from this project, not overlapping with the present report, have been published elsewhere (16, 17).

### Subjects

The subject enrollment and assessment protocols were approved by the institutional review boards of Weill Cornell Medical College (WCMC) in New York, and Stanford University in California. In collaboration with school, university, and community athletic organizations in respective local areas, middle-school through adult athletes were enrolled and baseline-assessed. For equity's purpose, athletes were enrolled independently of the level of contact involved in their participating sports. The inclusion criteria were participation in organized competitive athletic activity, being of age 12–30 years, normal or corrected-to-normal vision, and for athletes over the age 18, a high school diploma or equivalent, or expected timely high school graduation. Prior to data collection, written informed consent by adult subjects, or legal guardians of minor subjects with the minors' assent, was obtained in accordance with the Declaration of Helsinki.

During the baseline consent process, the athletes were given the option of allowing their athletic director, trainer, coach, or school nurse to contact the researchers if they sustained a concussion, as well as the research staff to contact the athletic staff to check on injury status. Acute post-concussion enrollment was based on inclusion criteria consisting of having experienced a concussion within 2 weeks that resulted in loss of consciousness, post-traumatic amnesia, dizziness, nausea, headaches, balance problems, blurred or double vision, or daze, and confusion, and on an exclusion criterion of intoxication at the time of injury. A diagnosis by a physician was not required. For the purpose of this report, we will identify athletes who were under 18 years of age when they were baseline-assessed as pediatric subjects.

Data were collected inside a parked recreational vehicle outfitted as a mobile testing site, or at the Citigroup Biomedical Imaging Center at WCMC. Data collection spanned from September of 2012 through September of 2016.

### Modified RPQ

The original form of the RPQ instructs the respondent to rate problems over the last 24 h relative to the premorbid levels (13). To assess the presence and severity of symptoms typically associated with concussion but instead in an everyday athletic setting, the instruction for baseline assessments was rephrased with the following sentence—“We would like to know if you have experienced any of the symptoms given below more than usual today or in the past week.” The original instruction was retained for post-concussion assessments. Further, for both implementations the “Additional Symptoms” section was replaced with three specific items: balance problems; feeling mentally “foggy;” and drowsiness. To sum up, there were a total of 19 items in the modified RPQ, with which we associated the following five subtypes of post-concussion clinical trajectories to determine the presence and severity of these symptom classes.

#### Cognitive-fatigue

We identified the four items from the RPQ, “fatigue, tiring more easily,” “forgetfulness, poor memory,” “poor concentration,” and “taking longer to think,” as being associated with the cognitive-fatigue profile. The presence of a severe, at-least-moderate, or at-least-mild symptom was identified if any of the four items was rated as 4, ≥3, or >0, respectively.

#### Vestibular

We identified the two items, “feeling of dizziness” and “balance problems,” as being associated with the vestibular profile. The presence of a severe, at-least-moderate, or at-least-mild symptom was identified if either of the two items was rated as 4, ≥3, or >0, respectively.

#### Oculomotor

We identified the two items, “blurred vision” and “double vision,” as being associated with the oculomotor profile. The presence of a severe, at-least-moderate, or at-least-mild symptom was identified if either of the two items was rated as 4, ≥3, or >0, respectively.

#### Anxiety/mood

We identified the four items, “being irritable, easily angered,” “feeling depressed or tearful,” “feeling frustrated or impatient,” and “restlessness,” as being associated with the anxiety/mood profile. The presence of a severe, at-least-moderate, or at-least-mild symptom was identified if any of the four items was rated as 4, ≥3, or >0, respectively.

#### Migraine

We identified the four items, “headaches,” “noise sensitivity, easily upset by loud noise,” “nausea and/or vomiting,” and “light sensitivity, easily upset by bright light,” as being associated with the migraine profile. The presence of a severe, at-least-moderate, or at-least-mild-symptom was identified if the headache item was rated as 4, ≥3, or >0 *and* in addition at least one of the other three items was rated as 4, ≥3, or >0, respectively.

No item from the RPQ was associated with the *cervical* profile/condition. In addition to the above five profiles, the presence of a severe, at-least-moderate, or at-least-mild symptom associated with *sleep disturbance* was identified if either “sleep disturbance” or “drowsiness” was rated as 4, ≥3, or >0, respectively. Thereby we specified a total of six symptom classes (five subtypes and one subtype-associated condition).

### Statistical analysis

A binomial test was used to contrast the prevalence of a specified attribute in the concussed cohort against the probability established by the baseline observation. A significant deviation from this probability was detected at the alpha level of 0.05. In addition, as described in the Results section below, being in the pediatric group and a history of a previous head injury (of unspecified severity) were both associated with a higher likelihood of getting concussed. To examine the association between these two attributes, we determined the odds ratio and its confidence interval.

## Results

Baseline assessments were conducted in a total of 3,091 qualified athletes. Three subjects had incomplete RPQ records. Of the remaining 3,088 subjects, 1,178 (38.2%) were female and 1,910 (61.9%) were male. There were 857 pediatric subjects (mean age: 15.3 years, SD: 1.4), of whom 317 (37.0%) were female and 540 were male (63.0%). A previous head injury was reported by 180 subjects of the pediatric group (21.0%) and 722 subjects of the adult group (32.4%), totaling 902 (29.2%).

After a baseline assessment, 89 subjects experienced a concussion and were re-assessed within 2 weeks of the injury, on average at 5.8 days post-injury (SD: 3.5). Consistent with the mostly random nature of concussive accidents, the distribution of timing of injuries since the baseline assessment closely followed a negative exponential pattern, with a time constant of ~3 months (mean: 3.1, SD: 2.9, range: 0–15). All 89 concussed subjects had valid RPQ records at both baseline and post-concussion time points. Of the 89, 38 subjects were in the pediatric group, within which 26 were male (68.4%) and 12 were female (31.6%), and 51 subjects were in the adult group, within which 22 were male (43.1%) and 29 were female (56.9%). Four of the concussed subjects in the pediatric group were over 18 years of age at the time of post-concussion assessment, but only by at most 5 months. A previous head injury was reported by 12 of the 38 concussed pediatric subjects (31.6%) and 27 of the 51 concussed adult subjects (52.9%), totaling 39 (29.2%).

**Figure 1:**
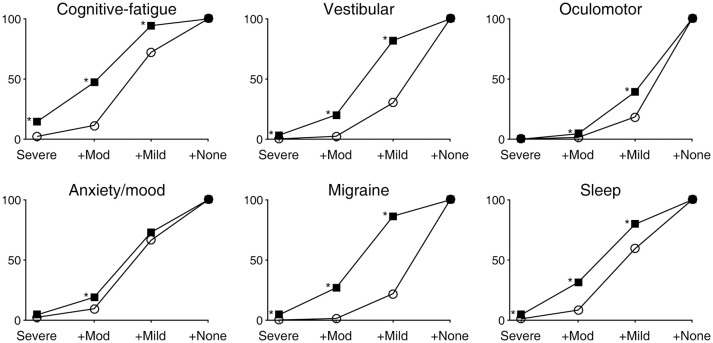
Prevalences of six classes of symptoms expressed as cumulative distributions. Open circles and filled squares indicate baseline and post-concussion findings, respectively. An asterisk indicates a statistically significant (*p* < 0.05) deviation of a post-concussion prevalence from the baseline-derived probability as determined by a binomial test. Also see Table 1.

The presence and severity of six classes of symptoms identified through combinations of RPQ items are summarized in Figure 1. In the plots, the data are expressed cumulatively; thus, the percentages associated with the labels +*Mod* and +*Mild* indicate those of at-least-moderate and at-least-mild symptom presentations, respectively. In all symptom classes, problems that were present at an at-least-moderate level were more prevalent after a concussion than at baseline (Table 1). One or more of the four RPQ items related to the cognitive-fatigue profile were most prevalently endorsed by the concussed subjects, with as much as 47.2% reporting at-least-moderate symptoms (57.9% in the pediatric subgroup and 39.2% in the adult subgroup). The two RPQ items related to the oculomotor profile were least commonly endorsed by the concussed subjects. We also noted that the prevalences of vestibular- and migraine-related symptoms at the at-least-mild level were more than twice elevated after a concussion.

**Table 1:** Prevalence of moderate-to-severe symptom identification.

	**Prevalence (%)**		p^*^
	**Concussed (*N* = 89)**	**Baseline (*N* = 3088)**	
Cognitive-fatigue	47.2	11.5	< 0.001
Vestibular	20.2	2.4	< 0.001
Oculomotor	4.5	1.5	0.042
Anxiety/mood	19.1	9.5	0.006
Migraine	27.0	1.4	< 0.001
Sleep	31.5	8.4	< 0.001

* *as determined by a binomial test*.

The identified post-concussion prevalence of at-least-moderate oculomotor symptoms was only marginally different from the probability derived from the baseline assessment (Table 1), and in the pediatric subgroup specifically, the prevalence at 2.6% was not statistically greater than the corresponding probability. In the pediatric subgroup, the identified prevalence of at-least-moderate anxiety/mood symptoms at 18.4% was also not statistically greater than the probability derived from the corresponding baseline assessment at 12.8%, which veered from the 9.5% overall and 8.2% adult probabilities. No substantial alteration was found between pediatric and adult subgroups in the prevalences of vestibular-, migraine-, or sleep-related symptoms.

We noted some sex-based symptom prevalence differences. All four subjects who reported severe migraine-related symptoms after a concussion were female (prevalence of 9.8 in female vs. 0% in male). Three of these subjects reported mild migraine-related symptoms at baseline while one was initially free of such symptoms. Similarly, all three subjects who reported severe vestibular-related symptoms after a concussion were female (prevalence of 7.3 in female vs. 0% in male). All these subjects reported mild vestibular-related symptoms at baseline. There was also a higher prevalence of at-least-mild sleep-related symptoms at baseline among female subjects (67.8 vs. 54.3% in male), which was further elevated after a concussion (92.7 vs. 68.8% in male). The trends were similar between pediatric and adult subgroups.

We next examined whether the prevalences of particular individual baseline attributes were different in those who experienced a concussion during the data collection period compared to the overall prevalences (Table 2). The sexes were not apportioned differently among the concussed subjects (53.9 male in the concussed vs. 61.9% male overall), but ages and previous histories of head injury were. In terms of baseline symptomatology, concussed subjects were more likely than typical to have endorsed cognitive-fatigue-, vestibular-, migraine-, and sleep-related items of the RPQ at varying levels.

**Table 2:** Baseline characteristics of those who became concussed during the study period compared against those of the entire sample.

	**Prevalence (%)**		p^*^
	**Concussed (*N* = 89)**	**Overall (*N* = 3088)**	
Pediatric age	42.7	27.8	0.003
Previous head injury	43.8	29.2	0.003
**COGNITIVE-FATIGUE**			
At least moderate	22.5	11.5	0.004
At least mild	83.1	72.2	0.024
**VESTIBULAR**			
At least mild	44.9	30.2	0.004
**MIGRAINE**			
At least moderate	4.5	1.4	0.039
At least mild	37.1	22.1	0.001
**SLEEP**			
Severe	4.5	1.1	0.019
At least moderate	15.7	8.4	0.020

* *as determined by a binomial test*.

As stated above, ages and previous histories of head injury were apportioned differently among the concussed subjects. We examined the possible association between age (pediatric vs. adult) and a previous head injury within the concussed group. The odds ratio was 0.41 with a confidence interval between 0.17 and 0.99, indicating that a concussed pediatric subject was less likely to have had a previous head injury than an adult counterpart.

**Figure 2:**
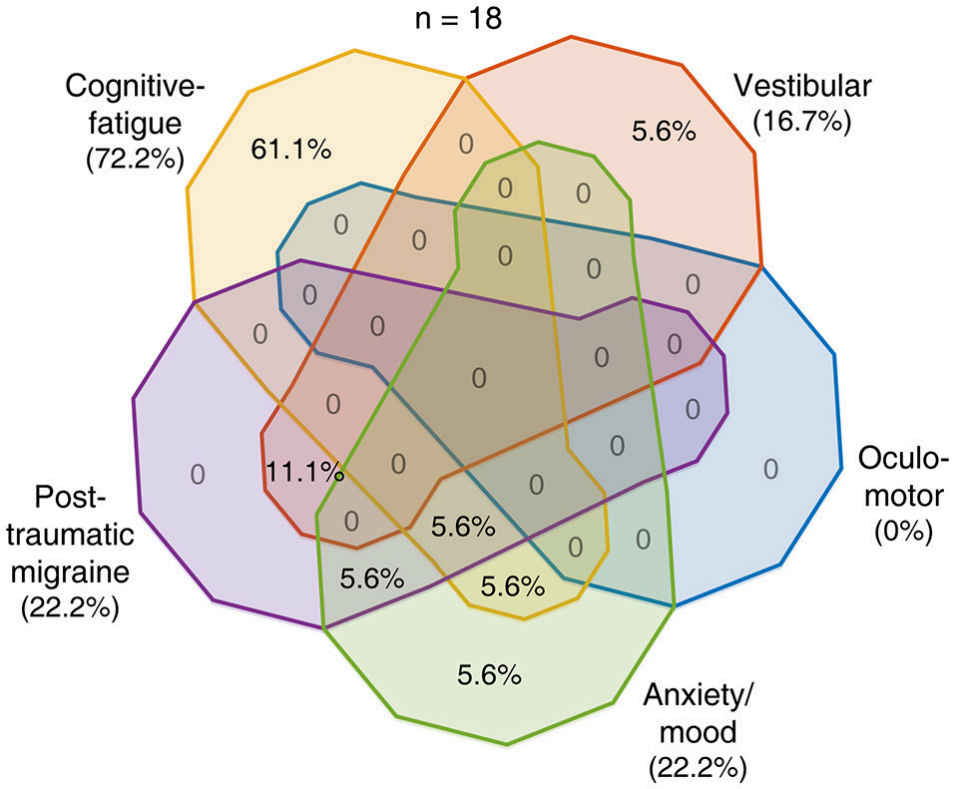
Overlaps in post-concussion profile characteristics identified with severe symptoms. Eighteen of 89 concussed subjects reported having a severe post-concussion symptom. The distribution of these 18 subjects among the various combinations of presences and absences of endorsement of the five symptom classes is shown as percentages in the Venn diagram with areas of overlap among the five pear-shaped regions.

**Figure 3:**
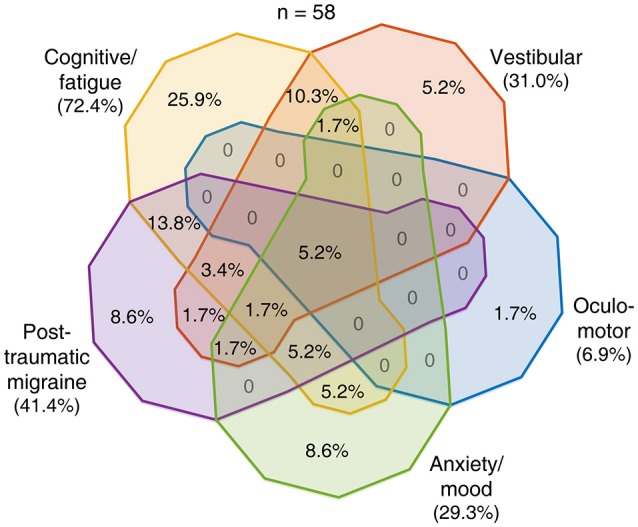
Overlaps in post-concussion profile characteristics identified with at-least-moderate symptoms. Fifty-seven of 89 concussed subjects reported having an at-least moderate post-concussion symptom. The distribution of these 57 subjects among the various combinations of presences and absences of endorsement of the five symptom classes is shown as percentages in the Venn diagram.

**Figure 4:**
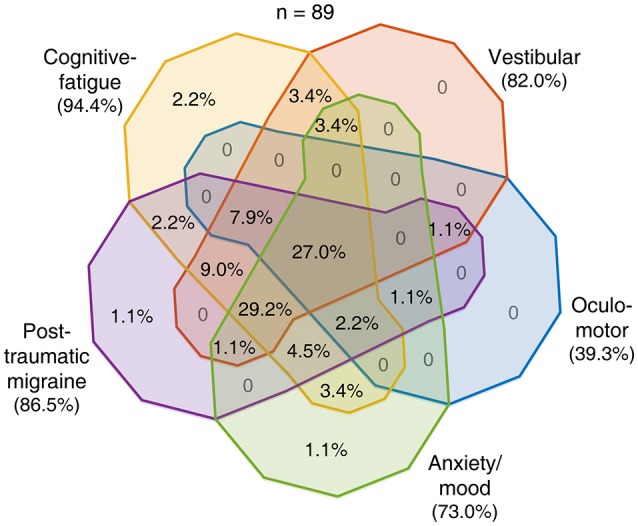
Overlaps in post-concussion profile characteristics identified with at-least mild symptoms. All 89 concussed subjects reported having an at-least mild post-concussion symptom. The distribution of these 89 subjects among the various combinations of presences and absences of endorsement of the five symptom classes is shown as percentages in the Venn diagram.

To evaluate the ability of the RPQ to delineate among the five profiles presumed to predict post-concussion trajectories, we examined similarities and differences with which the subjects endorsed RPQ items that we associated with these profiles. We constructed 31 logical relationships regarding the presence or absence of endorsement of each symptom class (excluding the absence of all) and sorted the subjects correspondingly (Figures 2–4). Given the size of compartmentalization, we did not subdivide the 89 concussed subjects by their baseline characteristics in this analysis. A total of 18 subjects reported severe post-concussion symptoms in the RPQ, of whom 13 (72.2%) reported those associated with the cognitive-fatigue profile, and 11 (61.1%) reported only such symptoms (Figure 2). The subjects reported few overlaps in severe symptoms, with all but one of the 18 subjects (94.4%) reporting at most two classes of symptoms with a severe rating. A total of 58 subjects reported at-leased-moderate post-concussion symptoms, of whom 42 (72.4%) reported those associated with the cognitive-fatigue profile (Figure 3). At this severity level also, the subjects reported relatively few overlaps in symptoms in general, with 47 of 58 subjects (81.0%) reporting at most two classes of symptoms with an at-least-moderate rating. All 89 concussed subjects reported at-least-mild post-concussion symptoms, of whom 59 (66.3%) reported symptoms with overlapping associations with at least four post-concussion profiles (Figure 4). Only 12 subjects (13.5%) reported symptoms with overlapping associations with at most two post-concussion profiles. Therefore, the RPQ may best identify patients' post-concussion profiles with moderate-to-severe increases in symptom severities.

## Discussion

The sample, represented by over 3,000 subjects at baseline, was a prospective cohort from a natural setting of competitive sports. Only 2.9% of the baseline-assessed athletes subsequently became concussed during the study period, likely reflecting the fact that the athletes were enrolled independently of the level of contact involved in their participating sports.

All concussed athletes in this study endorsed having some elevated symptoms in the RPQ. Although symptoms covered by the RPQ can appear in a normal setting as we also found, endorsements of these symptoms were measurably higher acutely following concussion. Among the six post-concussion symptom classes described by clinical and anecdotal evidence (5, 6, 11) and identifiable with the current implementation of the RPQ, cognitive-fatigue-related symptoms were most prevalently endorsed by concussed athletes at all levels of severity. Post-concussion increases in the prevalences of mild vestibular- and migraine-related symptoms were likewise substantial.

Symptoms related to the oculomotor profile were least prevalently endorsed through the RPQ by concussed athletes. This finding diverges from those derived from objective measures (18–21). It is possible that the low endorsement rate reflected a low representation of this profile in the study cohort. However, since the oculomotor profile were identified with only “blurred vision” and “double vision,” the low endorsement rate may be better explained by the limitation of the scope of the questionnaire. Therefore, the RPQ may be supplemented with additional items such as difficulty in reading/near work and in judging distances. Similarly, the RPQ contained no item related to the cervical profile, and it may be beneficial to supplement it with additional items such as neck pain and neck/upper extremity weakness.

Adult and pediatric athletes demonstrated generally similar symptomatology both at baseline and post-concussion. The pediatric athletes had comparatively elevated anxiety/mood problems at baseline, but such symptoms at a normal or subclinical level are common in adolescence (22, 23). Our pediatric athletes had a higher rate of concussion than adult athletes. Although age may play a role in concussion risk, there are contrasting findings in the literature (24). We also found some sex-based differences in symptomatology. Since our sample size and scope of collected data do not warrant a further analysis, the basis for the tendency we found is unclear. Age and a history of head injury were associated with a subsequent concussion. These two factors were related such that concussed adult athletes were more likely to have had a previous head injury than concussed pediatric athletes. This relation may be explained by a cumulative effect of concussion—adults are more likely to have had a previous head injury than youths, and a previous head injury can increase the chance of another concussion (24, 25).

Despite being a subjective measure and possibly limited in scope, the RPQ, in addition to its original purpose, may have the ability to delineate concussion profiles. In particular, among the five prescribed subtypes, there was a “sweet spot” in at-least-moderate symptom reports, which were endorsed by the majority of the concussed athletes and at the same time were observed within individuals with relatively few overlaps among different symptom classes. On the other hand, most concussed athletes reported at-least-mild problems of many different symptom classes. Therefore, moderate-to-severe increases in symptom severities as detected by the RPQ, when combined with objective measures, may well project patients' individual clinical trajectories and guide appropriate treatment approaches that maximize the treatment impact.

We also found that, in characterizing baseline RPQ responses, pre-existing symptoms that increase the chance of concussion may be identified, namely cognitive-fatigue-, vestibular-, migraine-, and sleep-related problems. Thus, in addition to a history of previous head injury, a well-known risk factor for concussion (24, 25), consideration of these symptoms may be integrated into concussion prevention strategies, such as promoting risk awareness and guiding the choice of activities. The female sex is also a recognized risk factor for sports concussion when comparisons between the sexes are made within specialties (24, 26, 27). However, this study did not identify a sex-based difference in concussion risk, possibly because we analyzed all sports collectively, the approach imposed by the protocol for subject selection.

## Conclusion

Classifying concussion in key subtypes according to presenting symptomatology at an early post-injury stage may allow prediction of clinical trajectories and delivery of targeted treatments. We characterized with a modified RPQ prevalences of concussion-related symptoms in baseline naturalistic and acute post-concussion contexts within an athletic setting. The sample size for those who became concussed was reduced to < 3% of the baseline. Although the RPQ is composed of simple self-report measures, symptom prevalences identified with it still rendered usefulness. That is, elevated symptoms detected with the RPQ within a week or so after a concussion may help identify patients' predicted clinical trajectories and guide care prioritization. The RPQ was still found to be limited in scope for such use, and thus supplementary question items may improve its ability. Finally, any interpretation must take place within the context of multiple measures, both subjective, and objective, applied to the evaluation of patients with concussion.

## Author contributions

JM and JG designed experiments and oversaw data collection. JM conducted the statistical analyses and drafted the manuscript. All authors contributed to the interpretation of data and to revising the work.

### Conflict of interest statement

JG is director of Sync-Think, Inc., and the inventor of U.S. patent 7,384,399. JM holds stock option in Sync-Think. JG and JM are inventors of pending patent applications PCT/US2014/050774, PCT/US2016/027923, and US15585057 potentially related to the subject matter described in this article. The remaining author declares that the research was conducted in the absence of any commercial or financial relationships that could be construed as a potential conflict of interest.

## References

[1] Panczykowski DM Pardini JE. The multidisciplinary concussion management program. Prog Neurol Surg. (2014) 28:195–212. 10.1159/000358780 24923404

[2] Collins MW Kontos AP Okonkwo DO Almquist J Bailes J Barisa M. Statements of agreement from the targeted evaluation and active management (TEAM) approaches to treating concussion meeting held in pittsburgh, October 15-16, 2015. Neurosurgery (2016) 79:912–29. 10.1227/NEU.0000000000001447 27741219 PMC5119544

[3] Ellis MJ Leddy J Willer B. Multi-disciplinary management of athletes with post-concussion syndrome: an evolving pathophysiological approach. Front Neurol. (2016) 7:136. 10.3389/fneur.2016.00136 27605923 PMC4995355

[4] Kenzie ES Parks EL Bigler ED Lim MM Chesnutt JC Wakeland W. Concussion as a multi-scale complex system: an interdisciplinary synthesis of current knowledge. Front Neurol. (2017) 8:513. 10.3389/fneur.2017.00513 29033888 PMC5626937

[5] Collins MW Kontos AP Reynolds E Murawski CD Fu FH. A comprehensive, targeted approach to the clinical care of athletes following sport-related concussion. Knee Surg Sports Traumatol Arthrosc. (2014) 22:235–46. 10.1007/s00167-013-2791-6 24337463

[6] Kontos AP Collins MW Concussion: A Clinical Profile Approach to Assessment and Treatment. American Psychological Assocation (2018). Washington, DC.

[7] Rathbone AT Tharmaradinam S Jiang S Rathbone MP Kumbhare DA. A review of the neuro- and systemic inflammatory responses in post concussion symptoms: introduction of the “post-inflammatory brain syndrome” PIBS. Brain Behav Immun. (2015) 46:1–16. 10.1016/j.bbi.2015.02.009 25736063

[8] Maruta J Lee SW Jacobs EF Ghajar J. A unified science of concussion. Ann N Y Acad Sci. (2010) 1208:58–66. 10.1111/j.1749-6632.2010.05695.x 20955326 PMC3021720

[9] Kenzie ES Parks EL Bigler ED Wright DW Lim MM Chesnutt JC. The dynamics of concussion: mapping pathophysiology, persistence, and recovery with causal-loop diagramming. Front Neurol. (2018) 9:203. 10.3389/fneur.2018.00203 29670568 PMC5893805

[10] Mathias JL Alvaro PK. Prevalence of sleep disturbances, disorders, and problems following traumatic brain injury: a meta-analysis. Sleep Med. (2012) 13:898–905. 10.1016/j.sleep.2012.04.006 22705246

[11] Broglio SP Collins MW Williams RM Mucha A Kontos AP. Current and emerging rehabilitation for concussion: a review of the evidence. Clin Sports Med. (2015) 34:213–31. 10.1016/j.csm.2014.12.005 25818710 PMC4387881

[12] Wickwire EM Williams SG Roth T Capaldi VF Jaffe M Moline M. Sleep, sleep disorders, and mild traumatic brain injury. What we know and what we need to know: findings from a national working group. Neurotherapeutics (2016) 13:403–17. 10.1007/s13311-016-0429-3 27002812 PMC4824019

[13] King NS Crawford S Wenden FJ Moss NE Wade DT. The rivermead post concussion symptoms questionnaire: a measure of symptoms commonly experienced after head injury and its reliability. J Neurol. (1995) 242:587–92. 10.1007/BF00868811 8551320

[14] Iverson GL Lange RT. Examination of “postconcussion-like” symptoms in a healthy sample. Appl Neuropsychol. (2003) 10:137–44. 10.1207/S15324826AN1003_02 12890639

[15] Ettenhofer ML Barry DM A comparison of long-term postconcussive symptoms between university students with and without a history of mild traumatic brain injury or orthopedic injury. J Int Neuropsychol Soc. (2012) 18:451–60. 10.1017/S1355617711001895 22321647

[16] Maruta J Spielman LA Rajashekar U Ghajar J. Visual tracking in development and aging. Front Neurol. (2017) 8:640. 10.3389/fneur.2017.00640 29250026 PMC5714854

[17] Maruta J Spielman LA Rajashekar U Ghajar J. Association of visual tracking metrics with post-concussion symptomatology. Front Neurol. (2018) 9:611. 10.3389/fneur.2018.00611 30093880 PMC6070608

[18] Ciuffreda KJ Ludlam DP Thiagarajan P Yadav NK Capo-Aponte J. Proposed objective visual system biomarkers for mild traumatic brain injury. Mil Med. (2014) 179:1212–7. 10.7205/MILMED-D-14-00059 25373043

[19] Maruta J Ghajar J. Detecting eye movement abnormalities from concussion. Prog Neurol Surg. (2014) 28:226–33. 10.1159/000358786 24923406

[20] Ventura RE Balcer LJ Galetta SL Rucker JC. Ocular motor assessment in concussion: current status and future directions. J Neurol Sci. (2016) 361:79–86. 10.1016/j.jns.2015.12.010 26810521

[21] Capó-Aponte JE Jorgensen-Wagers KL Sosa JA Walsh DV Goodrich GL Temme LA. Visual dysfunctions at different stages after blast and non-blast mild traumatic brain injury. Optom Vis Sci. (2017) 94:7–15. 10.1097/OPX.0000000000000825 26889821

[22] Bell-Dolan DJ Last CG Strauss CC. Symptoms of anxiety disorders in normal children. J Am Acad Child Adolesc Psychiatry (1990) 29:759–65. 10.1097/00004583-199009000-00014 2228930

[23] Beesdo K Knappe S Pine DS. Anxiety and anxiety disorders in children and adolescents: developmental issues and implications for DSM-V. Psychiatr Clin North Am. (2009) 32:483–524. 10.1016/j.psc.2009.06.002 19716988 PMC3018839

[24] Abrahams S Fie SM Patricios J Posthumus M September AV. Risk factors for sports concussion: an evidence-based systematic review. Br J Sports Med. (2014) 48:91–7. 10.1136/bjsports-2013-092734 24052371

[25] Guskiewicz KM McCrea M Marshall SW Cantu RC Randolph C Barr W. Cumulative effects associated with recurrent concussion in collegiate football players: the NCAA concussion study. JAMA (2003) 290:2549–55. 10.1001/jama.290.19.2549 14625331

[26] Covassin T Swanik CB Sachs ML. Sex differences and the incidence of concussions among collegiate athletes. J Athl Train. (2003) 38:238–44. 14608434 PMC233178

[27] Lincoln AE Caswell SV Almquist JL Dunn RE Norris JB Hinton RY. Trends in concussion incidence in high school sports: a prospective 11-year study. Am J Sports Med. (2011) 39:958–63. 10.1177/0363546510392326 21278427

